# Efficient combination of Human Papillomavirus Genotyping for the triage of women with Atypical Squamous Cells of Undetermined Significance in Chinese rural population: A population-based study

**DOI:** 10.7150/jca.55771

**Published:** 2021-03-14

**Authors:** Wei Wang, Huina Zhang, Leqian Lin, Aimin Yang, Jing Yang, Weihong Zhao, Zhilian Wang, Lili Zhang, Xiaoqiang Su, Zhe Wang, Chen Wang, Haitao Zhang, Bo Feng, Dongyan Li, Huiqiang Liu, Xiaofen Niu, Jintao Wang, Jinghui Song, Li Li, Weiguo Lv, Chengquan Zhao, Min Hao

**Affiliations:** 1 Department of Obstetrics and Gynecology, Second Hospital of Shanxi Medical University, Taiyuan, Shanxi 030001, China.; 2 Department of Pathology, University of Rochester Medical Center, Rochester, NY 14642, USA.; 3 Department of Medicine and Therapeutics, The Chinese University of Hong Kong, Prince of Wales Hospital, Hong Kong SAR, China.; 4 Hong Kong Institute of Diabetes and Obesity, The Chinese University of Hong Kong, Hong Kong SAR, China.; 5 Department of pathology, Second Hospital of Shanxi Medical University, Taiyuan, Shanxi 030001, China.; 6 Department of Epidemiology, School of Public Health, Shanxi Medical University, Taiyuan, Shanxi 030001, China.; 7 Department of Obstetrics and Gynecology, Affiliated Hospital of Inner Mongolia, Medical University, Huhhot 010000, China.; 8 Department of Obstetrics and Gynecology, Affiliated Tumor Hospital of Guangxi, Medical University, Nanning 530000, China.; 9 Department of Gynecologic Oncology, Women's Hospital, School of Medicine, Zhejiang University, Hangzhou, Zhejiang 310006, China.; 10 Department of Pathology, University of Pittsburgh Medical Center, Pittsburgh, PA 16066, USA.

**Keywords:** Human Papillomavirus (HPV) genotyping, Atypical Squamous Cells of Undetermined Significance (ASC-US), cervical cancer screening, Pap test, cervical intraepithelial neoplasia

## Abstract

**Objective:** In this prospective, population-based study, we evaluated the utility of high-risk human papillomavirus (HR-HPV) genotyping for triaging women with atypical squamous cells of undetermined significance (ASC-US) in the Chinese rural area.

**Methods:** A total of 40,000 women were recruited from rural areas of Shanxi Province, China, between June 2014 and December 2014. Women with Pap results of ASC-US underwent HPV genotyping, colposcopy and histopathological examination. For those with normal cervixes or cervical intraepithelial neoplasia (CIN) 1 on the initial evaluation, a 2-year follow-up study was performed.

**Results:** The reporting rate of ASC-US was 5.76% (2,304/40,000) in the study population. The detection rates of CIN 2 or above (CIN2+) and CIN 3 or above (CIN3+) in women with ASC-US were 7.28% and 1.75%, respectively. HPV 16 (39.53%), HPV 58 (17.83%), and HPV 52 (15.50%) were the three most prevalent HR-HPV genotypes among all women with ASC-US cytology. The five most common HR-HPV genotypes in CIN3+ lesions were HPV16, HPV58, HPV33, HPV31 and HPV18. Compared with the 15 HR-HPV testing, genotyping for a combination of HPV16/18/31/33/58 increased specificity significantly with virtually no loss of sensitivity for detecting CIN2+ and CIN3+ lesions, as well as significantly reduced colposcopy referral rate (23.15% vs 33.70%, p<0.01). In addition, in the 2-year follow-up period, women with infection of HPV16, 18, 31, 33 or 58 genotypes were the most likely population (92%, 23/25) to develop CIN2 lesion.

**Conclusion:** Our results demonstrate that genotyping for a combination of HPV16/18/31/33/58 provides a more efficient and cost-effective model to risk-stratify women with ASC-US in the Chinese rural population.

## Introduction

Atypical squamous cells of undetermined significance (ASC-US) cytology is the most frequent abnormal interpretation on cervical Papanicolaou (Pap) test, accounting for ~5% of all cervical cytology in the western population 1-3 and 3-5% in Chinese population 4-6. The management of women with ASC-US remains a clinical challenge, due to the equivocal cytology and variable underlying process from human papillomavirus (HPV)-unrelated, non-neoplastic conditions to the HPV-related cervical intraepithelial neoplasia (CIN) and carcinomas. Earlier studies have found that 3-36% of women with ASC-US cytology will have underlying CIN2 or more severe lesion (CIN2+) 7-9, and only a small percentage of ASC-US associated with cervical intraepithelial neoplasia 3 or worse (CIN3+) can be detected by follow-up colposcopic examination. Therefore, many efforts have been made trying to identify those who have cancer and significant precancerous lesions among women with ASC-US cytology, in order to improve the efficacy of screening and reduce the number of unnecessary colposcopic examination.

Based on the published evidence, the American Society for Colposcopy and Cervical Pathology (ASCCP) Guidelines have recommended women with an ASC-US cytology result should have a reflex HPV testing, and an immediate colposcopy referral or conservative follow-up based on patients' previous screening history is recommended for women with high-risk (HR) HPV-positive ASC-US 10, 11. Currently, the widely-used, US Food and Drug administration (FDA)-approved HPV testing platforms are largely pooled HR-HPV tests, which include 13 or 14 of the most common high-risk genotypes (16, 18, 31, 33, 35, 39, 45, 51, 52, 56, 58, 59, 68 and/or 66). However, the risk of progression to cervical cancer and CIN varies substantially among individual carcinogenic genotypes 12, 13. It has been reported that women with HPV16 or HPV18 positive ASC-US had approximately twice the risk of CIN3+ as women with ASC-US and high-risk HPV types other than 16 and 18 2, 14, 15. Thus, type-specific HPV identification appears to be a reasonable strategy to improve risk stratification of women with ASC-US and to reduce the colposcopy burden. This approach has been supported by the launches of additional HPV genotype 16, 18 and /or 45 in the recent FDA-approved Cobas 4800, Aptima and Onclarity tests.

It is well-known that the HR-HPV prevalence and distribution varies geographically worldwide. There are some distinctive characteristics in the Chinese population, including more frequent HPV52 and HPV58 genotypes, and less HPV45 and HPV18 genotypes in cervical cancer and precancerous lesions 16. In order to evaluate whether selection of certain HPV genotypes may be more efficient and cost-effective for the risk management of women with ASC-US in the Chinese rural population, in this study, we report the results of baseline and 2-year follow up of HPV genotyping and histologic correlation in 2,304 women with ASC-US cytology, from a large population-based cervical cancer screening program and a prospective cohort in the rural areas of Shanxi province, China.

## Materials and Methods

### Study population

The study design and methods have been described previously 17, 18. Briefly, we conducted free cervical cancer screening tests for eligible women who were permanently residing in two counties (Yangqu and Jiexiu) of Shanxi Province between June 2014 and December 2014. A total of 40,000 women aged 19-65 years completed an epidemiological questionnaire on potential risk factors for CIN and the Pap test. Women with Pap results of ASC-US underwent HPV genotyping, colposcopy and histopathological examination.

**Figure 1:**
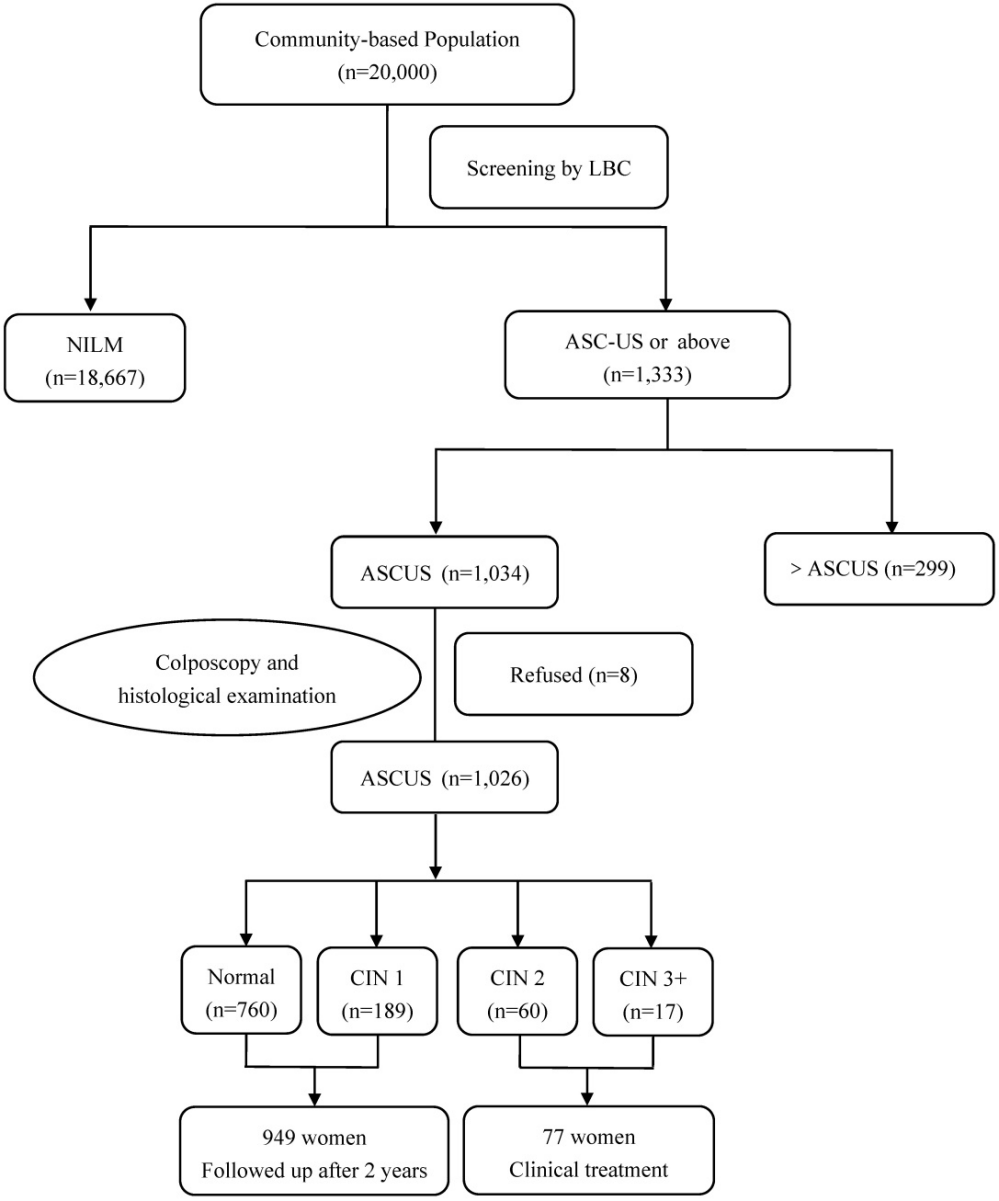
** Flow diagram of participants in Yangqu County.** Abbreviations: ASC-US, atypical squamous cells of undetermined significance; CIN, cervical intraepithelial neoplasia; HPV, human papillomavirus; LBC, liquid based cytology; NILM, negative for intraepithelial lesion or malignancy.

In Yangqu County, 1,034 women with ASC-US cytology were identified and 1,026 women underwent HPV baseline genotyping and immediate histopathologic examination. 77 women were diagnosed with CIN2 or worse including 4 squamous cell carcinomas on the histologic examination and all these patients received clinical treatment. 949 women were found to have normal cervixes or CIN1 and were followed-up for additional 2 years (**Figure 1**).

**Figure 2:**
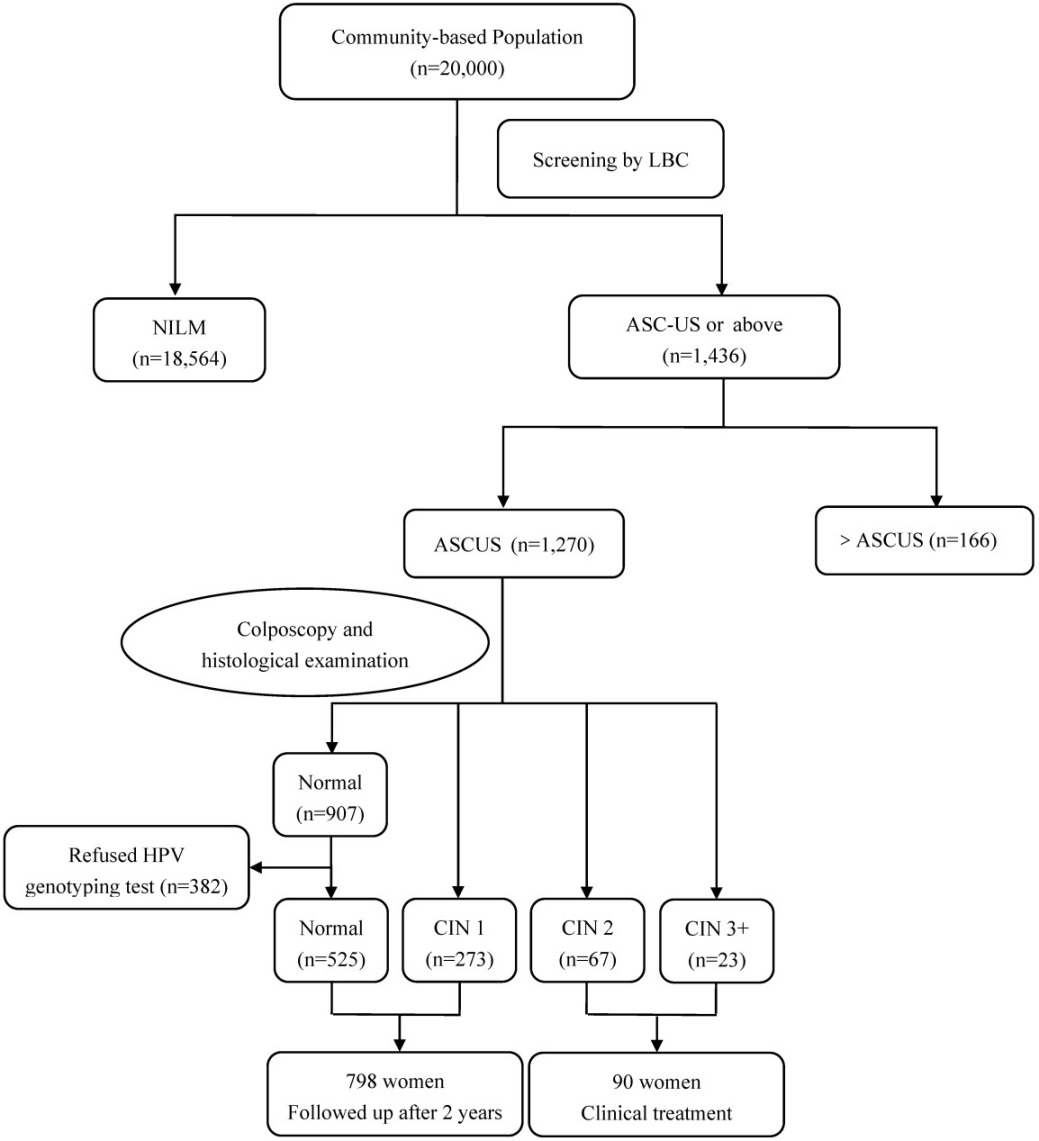
** Flow diagram of participants in Jiexiu County.** Abbreviations: ASC-US, atypical squamous cells of undetermined significance; CIN, cervical intraepithelial neoplasia; HPV, human papillomavirus; LBC, liquid based cytology; NILM, negative for intraepithelial lesion or malignancy.

Among 1,270 women with ASC-US cytology in Jiexiu County, 382 patients with normal cervix refused to have HPV genotyping test. 90 women with CIN2 or worse lesions received clinical treatment, while 798 women with normal or CIN1 lesion were followed-up for additional 2 years (**Figure 2**). All inspections and detections were implemented under double-blind conditions. The study was registered in the Chinese Clinical Trial Register (ChiCTR), registration number: ChiCTR-ROC-15006479. The study protocol and informed consent form were reviewed and approved by the Ethics Committee of Second Hospital of Shanxi Medical University.

### Cytology testing

The liquid-based cytology (LBC) method was used in this study. The LBC preparation was produced by Lituo Biotechnology Corp. (Hunan, China; http://www.lituo.com. cn). The cytological evaluation was performed by two cytopathologists at The Second Hospital of Shanxi Medical University. Cytology results were classified using the 2001 TBS criteria for reporting cervical cytology 19. The cases with an interpretation of ASC-US or a worse condition were further reviewed by a senior cytopathologist who was blinded to the results. Ten percent of cases with a negative report were reviewed by another pathologist to provide quality control.

### HPV genotyping

HPV genotyping was performed on the residual Pap test specimens using flow-through hybridization and a HybriMax gene chip (HybriBio Biotechnology Ltd, China). HybriBio GenoArray test is a commercially-available HPV genotyping diagnostic assay, and was approved by Conformitè Europèenne In Vitro Diagnostic (CE-IVD). Earlier studies have demonstrated the overall agreement in HR-HPV detection between HybriBio GenoArray genotyping assay and HC2 was 92.5-92.9% with a kappa value of 0.814 and 0.80 20, 21. All detection procedures were performed according to the manufacturer's instructions, as described previously 22. Twenty-one HPV genotypes were included in this testing platform, including 15 HR-HPV genotypes (HPV16, 18, 31,33, 35, 39, 45, 51, 52, 53, 56, 58, 59, 66 and 68) and 6 low-risk HPV (LR-HPV) genotypes (HPV6, 11, 42, 43, 44 and CP8304 (81)).

### Colposcopy and histological examination

The colposcopy procedure and biopsy were performed by experienced gynecological specialists from The Second Hospital of Shanxi Medical University. The average time from Pap sampling to colposcopy was 15 days (range: 3-62 days). Colposcopy examinations were performed using the Preventive Oncology International microbiopsy protocol. During the colposcopy, the cervix was divided into quadrants and each quadrant was examined independently. All visually abnormal areas were biopsied. Quadrants with normal colposcopic impressions were biopsied at the squamocolumnar junction (“random biopsy”). Women with abnormal cytology results and negative or inadequate colposcopic findings underwent endocervical curettage (ECC). All histological slides were reviewed by two gynecological pathologists, who were blinded to the cytology results at The Second Hospital of Shanxi Medical University. Immunohistochemistry for p16INK4A (p16) (ZM-0205, ZSGB-BIO, Peking, China) was utilized in adjudicating the diagnosis. If the two pathologists disagreed on the diagnosis, a third senior pathologist reviewed the slides, and some difficult or equivocal cases were considered for the pathology panel consensus diagnosis.

### Statistical analysis

The statistical analysis was performed using the Statistical Package for the Social Sciences (SPSS) version 22.0 software for windows (SPSS Inc., Chicago, IL, USA). Chi-squared test or Fisher's exact test were used for the categorical variables. Statistical tests were two-sided, and P <0.05 was considered statistically significant.

## Results

### HPV prevalence in women with ASC-US Pap test

Among 40,000 women in this study population, the overall ASC-US rate was 5.76% (2,304/40,000), including 5.17% (1,034/20,000) in Yangqu County and 6.35% (1,270/20,000) in Jiexiu County. Of 2,304 women with ASC-US cytology, 2,296 women underwent colposcopy and immediate histopathologic examination. Histology confirmed that 1,667 patients (72.60%) had normal cervixes, 462 (20.12%) had CIN1, 127 (5.53%) had CIN2, 27 (1.18%) had CIN3, and 13 (0.57%) had squamous cell carcinoma (SCC). The detection rates of CIN2+ and CIN3+ in women with Pap results of ASC-US were 7.28% and 1.75%, respectively.

Of 2,296 women with histologic examination, 1,914 women had HPV genotyping test. **Table 1** shows the relative frequencies of HPV infections in women with ASC-US Pap test according to histological diagnosis. The overall rates of HR-HPV and LR-HPV infections were 33.70% (645/1,914) and 1.83% (35/1,914). Among 645 women with HR-HPV infection, 445 patients were infected with a single HPV genotype and 200 patients were infected with multiple HPV genotypes including 20 patients co-infected with low-risk HPV genotypes. The prevalence of HR-HPV genotypes in women diagnosed with <CIN2, CIN2, CIN3 and SCC was 29.36%, 73.23%, 96.30% and 100%, respectively. LR-HPV infections alone, without HR-HPV infections, were not detected in any of the CIN3 and above cases.

The median age of women with ASC-US was 51 years (range: 20-65 years) in Yangqu County and 50 years (range: 20-65 years) in Jiexiu County. **Table 2** presents age-stratified HR-HPV prevalence in women with ASC-US cytology. Among all age groups, women aged between 30 and 39 years had peak rate of HR-HPV infection in Yangqu (58.2%, 71/122) and Jiexiu (31.4%, 38/121) Counties. There was a dramatic decline in the HR-HPV infection rates after age 50 in both Counties.

### Prevalence of different HR-HPV genotypes in women with ASC-US Pap test

In women with multiple infections, HPV genotypes were reported separately. The prevalence of HR-HPV genotypes in HR-HPV-positive cervical lesions in Yangqu County are presented in **Table 3A.** HPV16 (35.57%) was the most common HR-HPV genotype, followed, in order of decreasing frequency, by HPV52 (19.65%), HPV58 (17.91%), HPV53 (8.21%), HPV56 (6.97%), and HPV33 (6.97%). Additionally, HPV18 (4.48%) was in the 11th position. The distribution of HR-HPV genotypes by histologic outcome was different. HPV16 showed an increasing prevalence with the severity of the histological diagnosis (30.88% in <CIN2, 54.35% in CIN2, and 81.25% in CIN3+). HPV58 was the second-most prevalent genotype in CIN2+ lesions and the third-most prevalent genotype in <CIN2 lesions.

Interestingly, the prevalence of specific HR-HPV genotypes in HR-HPV-positive cervical lesions in Jiexiu County showed some difference from those seen in Yangqu county (**Table 3B**). The six most common HR-HPV genotypes in women with ASC-US Pap test, in decreasing order, were HPV16 (46.09%), HPV58 (17.70%), HPV51 (9.47%), HPV52 (8.64%), HPV33 (7.41%) and HPV31 (6.58%). HPV16 was still the most common genotypes in different histologic types. HPV58 and HPV33 were tied for the second-most prevalent genotypes in CIN2+ lesions.

**Table 3C** presents the distribution of HR-HPV genotypes in all the tested women in this cohort. HPV 16 (39.53%), HPV 58 (17.83%), and HPV 52 (15.50%) were the three most prevalent HR-HPV genotypes, while HPV 18 was less prevalent (4.34%). The five most common HR-HPV genotypes in women with CIN3+ lesions were HPV16 (82.05%), HPV58 (12.82%), HPV33 (10.26%), HPV31 (10.26%) and HPV18 (5.13%). The rest of 10 HR-HPV genotypes were found in ~17.94% of women with CIN3+ on the immediate histologic examination. In women with CIN2 lesions, the five most common HR-HPV genotypes were HPV16, HPV58, HPV31, HPV33 and HPV52. HPV18 was the seventh-most prevalent genotype in CIN2 lesions and the fifth-most prevalent genotype in CIN3+ lesions.

### Triage accuracy of different HPV genotypes models for women with ASC-US cytology

**Table 4** displays the sensitivity, specificity, PPV and NPV of the combination of 5 highest-risk HPV genotypes (HPV16/18/31/33/58) and 15 HR-HPV genotypes (HPV16/18/31/33/35/39/45/51/52/53/56/58/59/66/68) in detecting CIN2+ and CIN3+ lesions in women with ASC-US. The sensitivity of the 5 highest-risk HPV genotypes (HPV16/18/31/33/58) for detecting CIN2+ and CIN3+ lesions was 72.46 % and 95.00%, respectively. The specificity for detecting CIN2+ and CIN3+ lesions was 81.57% and 78.39%, respectively. The NPV for detecting CIN2+ and CIN3+ was 96.87% and 99.86%, respectively. The sensitivity and NPV of the 5 highest-risk HPV genotypes were similar to those of the 15 HR-HPV genotypes for detecting CIN2+ and CIN3+ lesions. However, compared with the 15 HR-HPV genotypes, the 5 highest-risk HPV genotypes had significantly higher specificity for detecting both CIN2+ and CIN3+ lesions. In addition, the 5 highest-risk HPV genotypes had significant higher PPV for detecting CIN2+ lesions. Moreover, the colposcopy referral rate using the 5 highest-risk HPV genotypes (23.15%, 443/1,914) was significantly lower than that using 15 HR-HPV genotypes (33.70%, 645/1,914).

### Two-year follow-up results of women with ASC-US cytology and initial baseline normal cervix or CIN1

Of 1,747 women with normal cervix or CIN1 at baseline colposcopic examination, 1,385 patients (79.28%) including 990 HR-HPV negative and 395 HR-HPV positive women underwent both HPV genotyping test and colposcopic examination at the 2-year follow-up. Among 990 women with initial negative HPV testing, 130 cases were found to have HR-HPV positive (13.13%) and 8 cases developed CIN2 at the 2-year follow-up. Among 395 women with baseline positive HPV testing, 50 cases had new HR-HPV infection (12.66%), and 52 cases had persistent infection (13.16%). 14 (26.9%, 14/52) women with persistent HR-HPV infection developed CIN2.

**Table 5** demonstrates the prevalence of HR-HPV genotypes of women who underwent 2-year follow-up testing including 180 women with new HR-HPV infection and 52 women with persistent HR-HPV infection. Among them, the most common genotypes were HPV16 (28.02%), HPV58 (20.26%) and HPV52 (17.67%). Of the 16 CIN2 cases with positive HR-HPV test, the 5 highest-risk HPV genotypes were HPV16 (68.75%), HPV58 (37.5%), HPV18 (12.5%), HPV31 (12.5%) and HPV33 (12.5%) (**Table 6**).

## Discussion

Cervical cancer remains an important public health issue in China, especially in the large rural areas where the health resources are limited, and the cervical cancer screening coverage is low. In order to develop a more cost-effective and efficient cervical cancer screening program in the Chinese rural population, we initiated a large population-based cervical cancer screening program and a prospective cohort in the rural areas of Shanxi province, China in 2014. We herein report the results of HPV genotyping, immediate histologic correlation and 2-year follow-up of 2,304 women with ASC-US cytology. To the best of our knowledge, this is the largest prospective study on ASC-US women with genotyping and histological correlation in the Chinese rural population.

In this study, the detection rate of ASC-US among 40,000 women in two rural counties was 5.76% (2,304/40,000), which is within the range of 3.7-10% of ASC-US reporting rate in the Chinese population after the introduction of LBC preparation method 4, 5, 23, 24. Our results showed approximately 33.70% of women with ASC-US were HR-HPV-positive, similar to the HR-HPV positive rate of 34.98% in the study of Zheng et al, but lower than those reported (48.7% and 49%) in other two Chinese studies 4, 23, 24. Similar to the findings in the study of Guo et al, the HR-HPV prevalence among women with ASC-US in this study was associated with the severity of cervical abnormalities 24. Furthermore, we also found that HPV 16, HPV58, HPV33, HPV31 and HPV18 were the five most prevalent HR-HPV genotypes associated with CIN3+ lesions in the study population. This finding was in general agreement with the results from previous studies 24-26. In the current investigation, the risks of CIN3+ lesions for HPV45 and HPV52 were not found in our cohort. Three patients with HPV45-infection had normal cervixes on the histopathologic examination. 89% of 100 women with HPV52 infection had normal or CIN1 findings and the rest of 11% had CIN2 lesion on the immediate histopathologic examination.

According to the principle of “equal management of equal risks”, clinical management should be treated differently for ASC-US patients with different HPV genotypes. Previous studies have confirmed that HR-HPV genotyping could help identify women at highest risk for high-grade cervical lesions 27-29. Through a systemic review, Bonde et al demonstrated that the published evidence from US, United Kingdom, Sweden, Denmark, and the Netherlands support the clinical utility for HPV genotyping in risk discrimination for CIN3+ lesions during cervical cancer screening^30^. There is very limited experience using HR-HPV genotyping in triaging Chinese women with ASC-US. Lin et al investigated the role of genotyping of HPV 16/18 in 329 Chinese women and found that the sensitivity and specificity for HPV16/18 in detecting CIN2+ lesion in women with ASC-US were 82% and 91% 31. Guo et al investigated 393 Chinese women with ASC-US cytology and found higher sensitivity of HPV16/18/33/52/58 (93%) in detecting CIN2+ in ASC-US cases compared with HPV16/18/52 (80%), HPV16/18/52/58 (89%), and the specificity of HPV16/18/33/52/58 for detecting CIN2+ was 76% 24. In the current study, we evaluated the possibility of using a combination of five most common HPV genotyping (HPV16/18/31/33/58), which were identified in patients with CIN3+ lesions in our study cohort, to risk-stratify the women with ASC-US. Our results showed that the 5 highest-risk HPV genotypes had a slightly lower sensitivity (72.46% vs 79.04% in detecting CIN2 and 95.0% vs 97.50% in detecting CIN3+), but significantly higher specificity (81.57% vs 70.64% for detecting CIN2+, and 78.36% vs 67.66% for detecting CIN3+, p<0.001) than 15 HR-HPV genotypes in detecting CIN3+ and CIN2+ lesions in women with ASC-US. In addition, the 5 highest-risk HPV genotypes also reduced the rate of colposcopy referrals by approximately 10% (p<0.001). Our current results, after analyzing more than 2,000 ASC-US cases support the strategy that genotyping of combination of the most common HR-HPV types would provide a more efficient and cost-effective model in risk-stratifying women with ASC-US.

In this study, we also followed-up the women with ASC-US cytology who did not receive any treatment for a 2-year period of time. Our results found that the incidence and risk of progression to CIN 2 were the highest in women with persistent HR-HPV infection during the follow-up period. These findings are in line with those of previous studies indicating that persistent HR-HPV infection is the most important risk factor for the development of cervical cancer and precancerous lesions 32-35. In the present study, women infected with those 5 highest-risk HPV genotypes were the most likely population to develop CIN2. As shown in another study, the 3-year cumulative risks of developing CIN3+ lesions for women with HPV16 (16.0%), HPV18 (7.4%), HPV31 (7.0%), and HPV33/HPV58 (7.1%) were higher than the overall 5.2% risk associated with Hybrid Capture 2 (HC2) positivity 36. These results further support the approach of genotyping for the combination of 5 highest-risk HPV genotypes could provide risk stratification for women with ASC-US. As mentioned earlier, due to the limited public health resource in the rural areas in China, the development of this specific HPV genotyping assay might significantly improve the cost-effectiveness of HPV testing by reducing unnecessary colposcopy burden as well as overtreatment, which would have significant impact in the Chinese rural population.

The current study has some limitations. First, this study population had not previously been vaccinated for HPV. As a result, the prevalence of HR-HPV genotypes in HPV-Vaccinated population may be different. Second, in this study, the selection of 5 HPV genotypes (HPV16/18/31/33/58) was based on a relatively small sample size, and larger sample sizes and study in different geographic regions are needed to validate this novel HPV genotyping assay.

In conclusion, in this large-scaled prospective study with cross-sectional analysis as well as 2-year follow-up analysis, the genotyping for a combination of HPV16/18/31/33/58 provides a more efficient and cost-effective risk-stratification model in women with ASC-US in our study population. Women who tested negative for the 5 highest-risk HPV genotypes (HPV16/18/31/33/58) may not require immediate colposcopy. Further prospective studies with larger sample sizes, as well as from different geographic regions are needed to validate this novel HPV genotyping assay in the triage of women with ASC-US in the Chinese rural population.

## Figures and Tables

**Table 1:** HPV prevalence in women with ASC-US cytology according to histologic diagnosis

Histology	Yangqu County			Jiexiu County			Total		
	No	HR-HPV n,%	LR-HPV n,%	No	HR-HPV n,%	LR-HPV n,%	No	HR-HPV n,%	LR-HPV n,%
<CIN2	949	340 (35.83)	19 (2.00)	798	173 (21.68)	11 (1.38)	1,747	513 (29.36)	30 (1.72)
CIN2	60	46 (76.67)	1 (1.67)	67	47 (70.15)	4 (5.97)	127	93 (73.23)	5 (3.94)
CIN3	13	12 (92.31)	0 (0.00)	14	14 (100.00)	0 (0.00)	27	26 (96.30)	0 (0.00)
SCC	4	4 (100.00)	0 (0.00)	9	9 (100.00)	0 (0.00)	13	13 (100.00)	0 (0.00)
Total	1,026	402 (39.18)	20 (1.95)	888	243 (27.36)	15 (1.69)	1,914	645 (33.70)	35 (1.83)

Note: 15 were co-infected with low-risk HPV genotypes (13 in <CIN2 and 1 in CIN2) in Yangqu County; 5 were co-infected with low-risk HPV genotypes (1 in <CIN2 and 4 in CIN2) in Yangqu County. Abbreviations: ASC-US, atypical squamous cell of-undetermined significance; CIN, cervical intraepithelial neoplasia; HR-HPV, high-risk human papillomavirus; LR-HPV, low-risk human papillomavirus; SCC, squamous cell carcinoma.

**Table 2:** Age-stratified HR-HPV prevalence in women with ASC-US cytology

Age	Yangqu			Jiexiu			Total		
	Case No	Positive	%	Case No	Positive	%	Case No	Positive	%
20-29	21	10	47.6	29	8	27.6	50	18	36.0
30-39	122	71	58.2	121	38	31.4	243	109	44.9
40-49	309	174	56.3	284	83	29.2	593	257	43.3
50-59	449	120	26.7	361	90	24.9	810	210	25.9
60-65	125	27	21.6	93	24	25.8	218	51	23.4
Total	1,026	402	39.2	888	243	27.4	1,914	645	33.7

Abbreviations: ASC-US, atypical squamous cells of undetermined significance.

**Table 3A:** Prevalence of HR-HPV genotypes in HR-HPV-positive cervical lesions in Yangqu County

HR-HPV genotypes	<CIN2 (n=340)		HR-HPV genotypes	CIN2 (n=46)		HR-HPV genotypes	CIN3+ (n=16)		HR-HPV genotypes	Total (n=402)	
	No	%		No	%		No	%		No	%
HPV16	105	30.88	HPV16	25	54.35	HPV16	13	81.25	HPV16	143	35.57
HPV52	72	21.18	HPV58	8	17.39	HPV58	3	18.75	HPV52	79	19.65
HPV58	61	17.94	HPV52	7	15.22	HPV31	2	12.50	HPV58	72	17.91
HPV53	33	9.71	HPV31	5	10.87	HPV33	2	12.50	HPV53	33	8.21
HPV56	23	6.76	HPV56	4	8.70	HPV18	1	6.25	HPV56	28	6.97
HPV66	22	6.47	HPV33	4	8.70	HPV56	1	6.25	HPV33	28	6.97
HPV33	21	6.18	HPV51	4	8.70	HPV66	1	6.25	HPV66	25	6.22
HPV51	20	5.88	HPV66	3	6.52	HPV35	1	6.25	HPV51	24	5.97
HPV68	20	5.88	HPV18	2	4.35	HPV52	0	0.00	HPV31	22	5.47
HPV18	15	4.41	HPV68	1	2.17	HPV53	0	0.00	HPV68	21	5.22
HPV31	15	4.41	HPV39	1	2.17	HPV51	0	0.00	HPV18	18	4.48
HPV39	14	4.12	HPV35	1	2.17	HPV68	0	0.00	HPV39	15	3.73
HPV59	14	4.12	HPV53	0	0.00	HPV39	0	0.00	HPV59	14	3.48
HPV35	11	3.24	HPV59	0	0.00	HPV59	0	0.00	HPV35	13	3.23
HPV45	2	0.59	HPV45	0	0.00	HPV45	0	0.00	HPV45	2	0.50

Abbreviations: ASC-US, atypical squamous cells of undetermined significance; CIN, cervical intraepithelial neoplasia; HR-HPV, high-risk human papillomavirus.

**Table 3B:** Prevalence of HR-HPV genotypes in HR-HPV-positive cervical lesions in Jiexiu County

HR-HPV genotypes	<CIN2 (n=173)		HR-HPV genotypes	CIN2 (n=47)		HR-HPV genotypes	CIN3+ (n=23)		HR-HPV genotypes	Total (n=243)	
	No	%		No	%		No	%		No	%
HPV16	69	39.88	HPV16	24	51.06	HPV16	19	82.61	HPV16	112	46.09
HPV58	33	19.08	HPV58	8	17.02	HPV58	2	8.70	HPV58	43	17.70
HPV51	19	10.98	HPV33	8	17.02	HPV31	2	8.70	HPV51	23	9.47
HPV52	17	9.83	HPV31	7	14.89	HPV33	2	8.70	HPV52	21	8.64
HPV39	14	8.09	HPV52	4	8.51	HPV53	2	8.70	HPV33	18	7.41
HPV68	10	5.78	HPV51	4	8.51	HPV18	1	4.35	HPV31	16	6.58
HPV53	10	5.78	HPV18	4	8.51	HPV59	1	4.35	HPV39	15	6.17
HPV66	8	4.62	HPV53	2	4.26	HPV66	1	4.35	HPV53	14	5.76
HPV33	8	4.62	HPV56	1	2.13	HPV39	0	0.00	HPV18	10	4.12
HPV31	7	4.05	HPV39	1	2.13	HPV45	0	0.00	HPV68	10	4.12
HPV18	5	2.89	HPV45	0	0.00	HPV51	0	0.00	HPV66	9	3.70
HPV56	4	2.31	HPV59	0	0.00	HPV52	0	0.00	HPV56	5	2.06
HPV59	3	1.73	HPV66	0	0.00	HPV56	0	0.00	HPV59	4	1.65
HPV45	1	0.58	HPV68	0	0.00	HPV68	0	0.00	HPV45	1	0.41
HPV35	0	0.00	HPV35	0	0.00	HPV35	0	0.00	HPV35	0	0.00

Abbreviations: ASC-US, atypical squamous cells of undetermined significance; CIN, cervical intraepithelial neoplasia; HR-HPV, high-risk human papillomavirus.

**Table 3C:** Prevalence of HR-HPV genotypes in HR-HPV-positive cervical lesions in the total study population

HR-HPV genotypes	<CIN2 (n=513)		HR-HPV genotypes	CIN2 (n=93)		HR-HPV genotypes	CIN3+ (n=39)		HR-HPV genotypes	Total (n=645)	
	No	%		No	%		No	%		No	%
HPV16	174	33.92	HPV16	49	52.69	HPV16	32	82.05	HPV16	255	39.53
HPV58	94	18.32	HPV58	16	17.20	HPV58	5	12.82	HPV58	115	17.83
HPV52	89	17.35	HPV31	12	12.90	HPV33	4	10.26	HPV52	100	15.50
HPV53	43	8.38	HPV33	12	12.90	HPV31	4	10.26	HPV53	47	7.29
HPV51	39	7.60	HPV52	11	11.83	HPV18	2	5.13	HPV51	47	7.29
HPV66	30	5.85	HPV51	8	8.60	HPV66	2	5.13	HPV33	46	7.13
HPV68	30	5.85	HPV18	6	6.45	HPV53	2	5.13	HPV31	38	5.89
HPV33	29	5.65	HPV56	5	5.38	HPV56	1	2.56	HPV66	34	5.27
HPV39	28	5.46	HPV66	3	3.23	HPV35	1	2.56	HPV56	33	5.12
HPV56	27	5.26	HPV39	2	2.15	HPV59	1	2.56	HPV68	31	4.81
HPV31	22	4.29	HPV53	2	2.15	HPV52	0	0.00	HPV39	30	4.65
HPV18	20	3.90	HPV68	1	1.08	HPV51	0	0.00	HPV18	28	4.34
HPV59	17	3.31	HPV35	1	1.08	HPV68	0	0.00	HPV59	18	2.79
HPV35	11	2.14	HPV59	0	0.00	HPV39	0	0.00	HPV35	13	2.02
HPV45	3	0.58	HPV45	0	0.00	HPV45	0	0.00	HPV45	3	0.47

Abbreviations: ASC-US, atypical squamous cells of undetermined significance; CIN, cervical intraepithelial neoplasia; HR-HPV, high-risk human papillomavirus.

**Table 4:** Performance for detecting CIN2+ and CIN3+ by 5 highest-risk HPV genotypes and 15 HR-HPV genotypes for all cases

Group	HPV assay	Sensitivity % (95% CI)	Specificity % (95% CI)	PPV % (95% CI)	NPV % (95% CI)	Referral rate (%)
CIN2+	5 highest-risk HPV genotypes	72.46 (64.92-78.94)	81.57 (79.65-83.34)	27.31 (23.27-31.76)	96.87 (95.82-97.68)	23.15 (443/1,914)
	15 HR-HPV genotypes	79.04 (71.93-84.79)	70.64 (68.43-72.75)	20.47 (17.46-23.83)	97.24 (96.14-98.04)	33.70 (645/1,914)
	P value	0.160	<0.001	0.009	0.570	<0.001
CIN3+	5 highest-risk HPV genotypes	95.00 (81.79-99.13)	78.39 (76.44-80.22)	8.58 (6.22-11.68)	99.86 (99.45-99.98)	23.15 (443/1,914)
	15 HR-HPV genotypes	97.50 (85.27-99.87)	67.66 (65.48-69.77)	6.05 (4.39-8.25)	99.99 (99.49-100.00)	33.70 (645/1,914)
	P value	1.000	<0.001	0.110	1.000	<0.001

Abbreviations: CI, confidence interval; CIN, cervical intraepithelial neoplasia; HPV, human papillomavirus; NPV, negative predictive value; PPV, positive predictive value.

**Table 5:** Prevalence of HR-HPV genotypes at 2 years of follow-up in women with initial ASC-US cytology and normal cervix and CIN1 on immediate histologic examination

HR-HPV genotypes	HR-HPV New infection (n=180)		HR-HPV genotypes	HR-HPV persistence (n=52)		HR-HPV genotypes	Total (n=232)	
	No	%		No	%		No	%
HPV16	46	25.56	HPV16	19	36.54	HPV16	65	28.02
HPV58	34	18.89	HPV58	13	25.00	HPV58	47	20.26
HPV52	31	17.22	HPV52	10	19.23	HPV52	41	17.67
HPV39	19	10.56	HPV53	5	9.62	HPV53	22	9.48
HPV51	18	10.00	HPV33	4	7.69	HPV39	20	8.62
HPV53	17	9.44	HPV18	3	5.77	HPV51	20	8.62
HPV33	14	7.78	HPV31	3	5.77	HPV33	18	7.76
HPV31	12	6.67	HPV51	2	3.85	HPV31	15	6.47
HPV66	10	5.56	HPV39	1	1.92	HPV18	11	4.74
HPV68	10	5.56	HPV68	1	1.92	HPV68	11	4.74
HPV18	8	4.44	HPV35	0	0.00	HPV66	10	4.31
HPV56	5	2.78	HPV45	0	0.00	HPV56	5	2.16
HPV35	4	2.22	HPV56	0	0.00	HPV35	4	1.72
HPV59	3	1.67	HPV59	0	0.00	HPV59	3	1.29
HPV45	2	1.11	HPV66	0	0.00	HPV45	2	0.86

Abbreviations: ASC-US, atypical squamous cells of undetermined significance; CIN, cervical intraepithelial neoplasia; HR-HPV, high-risk human papillomavirus.

**Table 6:** Prevalence of HR-HPV genotypes in HR-HPV-positive cervical lesions at 2 years of follow-up in women with initial ASC-US cytology and normal cervix and on immediate histologic examination

HR-HPV genotypes	<CIN2 (n=216)		HR-HPV genotypes	CIN2 (n=16)		HR-HPV genotypes	Total (n=232)	
	No	%		No	%		No	%
HPV16	44	20.37	HPV16	11	68.75	HPV16	55	23.71
HPV58	33	15.28	HPV58	6	37.5	HPV58	39	16.81
HPV52	31	14.35	HPV18	2	12.5	HPV52	32	13.79
HPV39	19	8.80	HPV31	2	12.5	HPV39	19	8.19
HPV51	18	8.33	HPV33	2	12.5	HPV51	18	7.76
HPV53	17	7.87	HPV52	1	6.25	HPV53	18	7.76
HPV33	14	6.48	HPV53	1	6.25	HPV33	16	6.90
HPV31	11	5.09	HPV35	0	0	HPV31	13	5.60
HPV66	10	4.63	HPV39	0	0	HPV18	10	4.31
HPV68	10	4.63	HPV45	0	0	HPV66	10	4.31
HPV18	8	3.70	HPV51	0	0	HPV68	10	4.31
HPV56	5	2.31	HPV56	0	0	HPV56	5	2.16
HPV35	4	1.85	HPV59	0	0	HPV35	4	1.72
HPV59	3	1.39	HPV66	0	0	HPV59	3	1.29
HPV45	2	0.93	HPV68	0	0	HPV45	2	0.86

Abbreviations: ASC-US, atypical squamous cells of undetermined significance; CIN, cervical intraepithelial neoplasia; HR-HPV, high-risk human papillomavirus.

## Abbreviations

ASC-US: atypical squamous cells of undetermined significance
CI: confidence interval
CIN: cervical intraepithelial neoplasia
ECC: endocervical curettage
HR-HPV: high-risk human papillomavirus
LBC: liquid based cytology
NPV: negative predictive value
PPV: positive predictive value
SCC: squamous cell carcinoma
TBS: The Bethesda system

## References

[1] Davey DD, Souers RJ, Goodrich K, Mody DR, Tabbara SO, Booth CN (2019). Bethesda 2014 Implementation and Human Papillomavirus Primary Screening: Practices of Laboratories Participating in the College of American Pathologists PAP Education Program. Archives of pathology & laboratory medicine.

[2] Stoler MH, Wright TC Jr, Sharma A, Apple R, Gutekunst K, Wright TL (2011). High-risk human papillomavirus testing in women with ASC-US cytology: results from the ATHENA HPV study. American journal of clinical pathology.

[3] College of American Pathologists, Cytopathology Checklist, Northwestern Memorial Hospital Laboratories. 2017; (CAP Number 1870801)

[4] Zheng B, Yang H, Li Z, You J, Wei G, Zhang H (2019). Atypical Squamous Cells of Undetermined Significance Cervical Cytology Report Rate and Histologic Follow-up Findings From the Largest College of American Pathologists-Certified Laboratory in China. Archives of pathology & laboratory medicine.

[5] Tao X, Austin RM, Kong L, Sun Q, Lv Q, Xu H (2019). Nationwide survey of cervical cytology laboratory practices in China. Journal of the American Society of Cytopathology.

[6] Tao X, Zhang H, Wang L, Pan Q, Ji S, Zhou X (2021). Atypical squamous cells of undetermined significance cervical cytology in the Chinese population: Age-stratified reporting rates, high-risk HPV testing, and immediate histologic correlation results. Cancer Cytopathol.

[7] Cox JT, Lorincz AT, Schiffman MH, Sherman ME, Cullen A, Kurman RJ (1995). Human papillomavirus testing by hybrid capture appears to be useful in triaging women with a cytologic diagnosis of atypical squamous cells of undetermined significance. American journal of obstetrics and gynecology.

[8] Wright TC, Sun XW, Koulos J (1995). Comparison of management algorithms for the evaluation of women with low-grade cytologic abnormalities. Obstetrics and gynecology.

[9] Kinney WK, Manos MM, Hurley LB, Ransley JE (1998). Where's the high-grade cervical neoplasia? The importance of minimally abnormal Papanicolaou diagnoses. Obstetrics and gynecology.

[10] Perkins RB, Guido RS, Castle PE, Chelmow D, Einstein MH, Garcia F (2020). 2019 ASCCP Risk-Based Management Consensus Guidelines for Abnormal Cervical Cancer Screening Tests and Cancer Precursors. Journal of Lower Genital Tract Disease.

[11] Massad LS, Einstein MH, Huh WK, Katki HA, Kinney WK, Schiffman M (2013). 2012 updated consensus guidelines for the management of abnormal cervical cancer screening tests and cancer precursors. Obstetrics and gynecology.

[12] Kjær SK, Frederiksen K, Munk C, Iftner T (2010). Long-term absolute risk of cervical intraepithelial neoplasia grade 3 or worse following human papillomavirus infection: role of persistence. Journal of the National Cancer Institute.

[13] Sand FL, Munk C, Frederiksen K, Junge J, Iftner T, Dehlendorff C (2019). Risk of CIN3 or worse with persistence of 13 individual oncogenic HPV types. International journal of cancer.

[14] Einstein MH, Martens MG, Garcia FA, Ferris DG, Mitchell AL, Day SP (2010). Clinical validation of the Cervista HPV HR and 16/18 genotyping tests for use in women with ASC-US cytology. Gynecologic oncology.

[15] Gage JC, Schiffman M, Solomon D, Wheeler CM, Castle PE (2010). Comparison of measurements of human papillomavirus persistence for postcolposcopic surveillance for cervical precancerous lesions. Cancer epidemiology, biomarkers & prevention: a publication of the American Association for Cancer Research, cosponsored by the American Society of Preventive Oncology.

[16] Zhou HL, Zhang W, Zhang CJ, Wang SM, Duan YC, Wang JX (2018). Prevalence and distribution of human papillomavirus genotypes in Chinese women between 1991 and 2016: A systematic review. The Journal of infection.

[17] Yang J, Yang A, Wang Z, Wang W, Wang Z, Wang Y (2018). Interactions between serum folate and human papillomavirus with cervical intraepithelial neoplasia risk in a Chinese population-based study. The American journal of clinical nutrition.

[18] Li X, Ding L, Song L, Gao W, Wang L, Wang J (2020). Effects of exposure to polycyclic aromatic hydrocarbons combined with high-risk human papillomavirus infection on cervical intraepithelial neoplasia: A population study in Shanxi Province, China. International journal of cancer.

[19] Solomon D, Davey D, Kurman R, Moriarty A, O'Connor D, Prey M (2002). The 2001 Bethesda System: terminology for reporting results of cervical cytology. Jama.

[20] Tao P, Zheng W, Wang Y, Bian ML (2012). Sensitive HPV genotyping based on the flow-through hybridization and gene chip. Journal of biomedicine & biotechnology.

[21] Zhang L, Lin Y, Li JK (2014). Concordance in cervical HPV detection between hybrid capture 2 and HPV GenoArray tests. Asian Pacific journal of cancer prevention: APJCP.

[22] Wang Y, Wang S, Shen J, Peng Y, Chen L, Mai R (2016). Genotype Distribution of Human Papillomavirus among Women with Cervical Cytological Abnormalities or Invasive Squamous Cell Carcinoma in a High-Incidence Area of Esophageal Carcinoma in China. BioMed research international.

[23] Tao X, Zhang H, Wang L, Pan Q, Ji S, Zhou X (2020). Atypical squamous cells of undetermined significance cervical cytology in the Chinese population: Age-stratified reporting rates, high-risk HPV testing, and immediate histologic correlation results. Cancer Cytopathol.

[24] Guo Z, Jia MM, Chen Q, Chen HM, Chen PP, Zhao DM (2019). Performance of Different Combination Models of High-Risk HPV Genotyping in Triaging Chinese Women With Atypical Squamous Cells of Undetermined Significance. Frontiers in oncology.

[25] Chen HC, You SL, Hsieh CY, Schiffman M, Lin CY, Pan MH (2011). Prevalence of genotype-specific human papillomavirus infection and cervical neoplasia in Taiwan: a community-based survey of 10,602 women. International journal of cancer.

[26] Chan PK, Ho WC, Chan MC, Wong MC, Yeung AC, Chor JS (2014). Meta-analysis on prevalence and attribution of human papillomavirus types 52 and 58 in cervical neoplasia worldwide. PloS one.

[27] Wright TC Jr, Stoler MH, Parvu V, Yanson K, Eckert K, Kodsi S (2019). Detection of Cervical Neoplasia by Human Papillomavirus Testing in an Atypical Squamous Cells-Undetermined Significance Population: Results of the Becton Dickinson Onclarity Trial. American journal of clinical pathology.

[28] Ge Y, Christensen P, Luna E, Armylagos D, Xu J, Schwartz MR (2019). Role of HPV genotyping in risk assessment among cytology diagnosis categories: analysis of 4562 cases with cytology-HPV cotesting and follow-up biopsies. Int J Gynecol Cancer.

[29] Dong L, Hu SY, Zhang Q, Feng RM, Zhang L, Zhao XL (2017). Risk Prediction of Cervical Cancer and Precancers by Type-Specific Human Papillomavirus: Evidence from a Population-Based Cohort Study in China. Cancer prevention research (Philadelphia, Pa).

[30] Bonde JH, Sandri MT, Gary DS, Andrews JC (2020). Clinical Utility of Human Papillomavirus Genotyping in Cervical Cancer Screening: A Systematic Review. J Low Genit Tract Dis.

[31] Lin CQ, Cui JF, Zhang X, Pan QJ, Chen W, Qiao YL (2015). Human Papillomavirus Genotyping to Predict the Risk of Cervical Precancerous Lesions or Cancer in Women with Minor Abnormal Cytology in China. Acta cytologica.

[32] Schlecht NF, Kulaga S, Robitaille J, Ferreira S, Santos M, Miyamura RA (2001). Persistent human papillomavirus infection as a predictor of cervical intraepithelial neoplasia. Jama.

[33] Koshiol J, Lindsay L, Pimenta JM, Poole C, Jenkins D, Smith JS (2008). Persistent human papillomavirus infection and cervical neoplasia: a systematic review and meta-analysis. American journal of epidemiology.

[34] Travassos AG, Netto E, Xavier-Souza E, Nóbrega I, Adami K, Timbó M (2017). Predictors of HPV incidence and clearance in a cohort of Brazilian HIV-infected women. PloS one.

[35] Park Y, Kim TJ, Hwang CS, Cho CH, Jeong DH, Seong SJ (2019). Risk of cervical dysplasia among human papillomavirus-infected women in Korea: a multicenter prospective study. Journal of gynecologic oncology.

[36] Schiffman M, Vaughan LM, Raine-Bennett TR, Castle PE, Katki HA, Gage JC (2015). A study of HPV typing for the management of HPV-positive ASC-US cervical cytologic results. Gynecologic oncology.

